# CHALLENGES AND SUPPORTS FOR VETERAN CAREGIVERS DURING THE COVID-19 PANDEMIC: A MIXED-METHODS STUDY

**DOI:** 10.1093/geroni/igac059.1147

**Published:** 2022-12-20

**Authors:** Lauren Penney, Polly Noel, Karla Hernandez-Swift, Luci Leykum, Ranak Trivedi, Stuti Dang, Andrea Kalvesmaki, Jorie Butler

**Affiliations:** South Texas Veterans Health Care System, San Antonio, Texas, United States; South Texas Veterans Health Care System, San Antonio, Texas, United States; Texas State University, San Antonio, Texas, United States; South Texas Veterans Health Care System, San Antonio, Texas, United States; Stanford University, Palo Alto, California, United States; Miami Veterans Affairs Healthcare System, Miami, Florida, United States; Informatics, Decision-Enhancement and Analytic Sciences Center, Salt Lake City, Utah, United States; Salt Lake City Veterans Health Care System, Salt Lake City, Utah, United States

## Abstract

Informal caregivers face challenges in supporting older or medically-complex Veterans, which could be exacerbated by the COVID-19 pandemic. Our mixed methods observational study explored Veteran caregivers’ supports, challenges, and self-identified impacts during the pandemic. Caregivers whose veterans needed help with at least one activity of daily living for the last year and received care at one of five Veterans Health Administration (VA) study sites were eligible. Survey participants (n=46) were 96% female, 32-83 years old (median 59); most (83%) cared for a spouse. A majority (67%, n=31) reported increased stress since the start of the pandemic. Top sources of increased stress included worry about COVID-19 infection, increased caregiving responsibility, delayed access to care, concerns about vaccine safety, and employment or financial concerns. Caregiver interviews (n=26) qualitatively analyzed using a rapid, templated approach identified the following themes: (1) the benefits and challenges of VA COVID precautions to Veteran care access (e.g. telehealth, getting care for new problems), (2) supports afforded by and limits of the expansion of the VA Caregiver Support Program, (3) declines in Veteran physical and cognitive functioning, (4) increased caregiver role in Veterans’ support and care, (5) changes in work and living situations to address increased caregiving needs and/or reduce risk of exposure, and (6) loss of and then return to more usual routines and social outlets amid ongoing COVID-related uncertainties. Recommendations include targeted, personalized outreach to engage caregivers in existing supports, removing barriers and streamlining processes for obtaining services, and creating durable caregiver-to-caregiver, peer support opportunities.

